# Multi-echo fMRI, resting-state connectivity, and high psychometric schizotypy

**DOI:** 10.1016/j.nicl.2018.11.013

**Published:** 2018-11-20

**Authors:** Maria Waltmann, Owen O'Daly, Alice Egerton, Katrina McMullen, Veena Kumari, Gareth J. Barker, Steve C.R. Williams, Gemma Modinos

**Affiliations:** a Department of Psychosis Studies, Institute of Psychiatry, Psychology & Neuroscience, King's College London, UK; b Department of Neuroimaging, Institute of Psychiatry, Psychology & Neuroscience, King's College London, UK; c Centre for Cognitive Neuroscience, College of Health and Life Sciences, Brunel University London, UK; d Department of Psychology, Institute of Psychiatry, Psychology & Neuroscience, King's College London, UK

**Keywords:** Multi-echo fMRI, Schizotypy, Psychosis, Functional connectivity, Striatum, Resting-state

## Abstract

Disrupted striatal functional connectivity is proposed to play a critical role in the development of psychotic symptoms. Previous resting-state functional magnetic resonance imaging (rs-fMRI) studies typically reported disrupted striatal connectivity in patients with psychosis and in individuals at clinical and genetic high risk of the disorder relative to healthy controls. This has not been widely studied in healthy individuals with subclinical psychotic-like experiences (schizotypy). Here we applied the emerging technology of multi-echo rs-fMRI to examine corticostriatal connectivity in this group, which is thought to drastically maximize physiological noise removal and increase BOLD contrast-to-noise ratio. Multi-echo rs-fMRI data (echo times, 12, 28, 44, 60 ms) were acquired from healthy individuals with low (LS, *n* = 20) and high (HS, *n* = 19) positive schizotypy as determined with the Oxford-Liverpool Inventory of Feelings and Experiences (O-LIFE). After preprocessing to ensure optimal contrast and removal of non-BOLD signal components, whole-brain functional connectivity from six striatal seeds was compared between the HS and LS groups. Effects were considered significant at cluster-level *p* < .05 family-wise error correction. Compared to LS, HS subjects showed lower rs-fMRI connectivity between ventromedial prefrontal regions and ventral striatal regions. Lower connectivity was also observed between the dorsal putamen and the hippocampus, occipital regions, as well as the cerebellum. These results demonstrate that subclinical positive psychotic-like experiences in healthy individuals are associated with striatal hypoconnectivity as detected using multi-echo rs-fMRI. Further application of this approach may aid in characterizing functional connectivity abnormalities across the extended psychosis phenotype.

## Highlights

• Multi-echo fMRI was used to investigate resting-state connectivity in subjects with low and high psychometric schizotypy (HS).
• High schizotypy subjects showed hypoconnectivity between ventral striatum and ventromedial prefrontal cortex.
• These subjects also showed hypoconnectivity between dorsal striatum and temporooccipital regions.

## 1 Introduction

Over the last two decades, the dysconnection hypothesis of schizophrenia (Friston et al., 2016; Friston and Frith, 1995; McGuire and Frith, 1996) has gained growing neurobiological support due to technical advances in structural and functional magnetic resonance imaging (Pettersson-Yeo et al., 2011). The dysconnection hypothesis suggests that the hallmark symptoms of schizophrenia arise from abnormal functional integration between distributed brain regions due to altered neuromodulation of synaptic plasticity, particularly in regions with dopaminergic afferents (Adams et al., 2013; Friston et al., 2016). A key area is the striatum, which receives prominent innervations from dopaminergic neurons in the midbrain, and is central to the orchestration of activity of limbic, associative and motor brain regions through interconnected cortico-striatal loops, thereby supporting a range of neural computations necessary for normal cognitive function (Haber, 2016). Striatal dysregulation may therefore be involved in widespread disruption of these circuits and the emergence of positive symptoms. Indeed, a number of studies in patients with schizophrenia and individuals at clinical high risk (CHR) of psychosis reported increased presynaptic dopamine synthesis capacity and release in the striatum (see Howes et al., 2012 for a meta-analysis), and a direct correlation between the extent of striatal dysfunction and the severity of positive psychotic symptoms in patients (Allen et al., 2016; Kindler et al., 2017; Sorg et al., 2013). Moreover, there is evidence to suggest that positive symptoms may be associated with disrupted task-related striatal activation and connectivity during the attribution of aberrant salience to otherwise irrelevant stimuli in healthy individuals, CHR subjects, and patients with a full-blown psychotic disorder (Boehme et al., 2015; Diaconescu et al., 2010; Pankow et al., 2016; Roiser et al., 2013; Schlagenhauf et al., 2009; Winton-Brown et al., 2017, Winton-Brown et al., 2014). Such evidence aligns well with predictions based on animal models of psychosis (Lodge and Grace, 2011) which show that striatal dysfunction may result from increased hippocampal activity, which may in turn be related to prefrontal cortex (PFC) abnormalities (Gomes and Grace, 2017), and propose that disrupted interactions within this corticostriatal circuit contribute to the development of aberrant salience processing and positive symptoms (Grace, 2016).

In this context, resting-state functional magnetic resonance imaging (rs-fMRI) provides a powerful tool to examine patterns of altered functional connectivity and their relationship to symptomatology in patients with established psychosis as well as in individuals at genetic or CHR of psychosis (e.g. Dandash et al., 2014; Fornito et al., 2013; Peters et al., 2017). rs-fMRI studies focusing on striatal connectivity in patients with schizophrenia and in their relatives have reported altered functional integration of this region with a number of cortical areas, including mainly the prefrontal and temporal cortices (Horga et al., 2016; Lin et al., 2018; Peters et al., 2017; Sole-Padulles et al., 2016; Tu et al., 2012). Dimensional views of psychosis postulate that there is continuity between subclinical psychotic-like experiences which can be detected in healthy people using validated self-report questionnaires (that is, schizotypy) and psychotic symptoms in patients with schizophrenia (Linscott and van Os, 2013; Nelson et al., 2013). Consistent with this psychosis continuum view, a recent rs-fMRI study reported that scores on the positive dimension of schizotypy (relating to cognitive-perceptual experiences) were positively associated with ventral striatal–PFC connectivity, and negatively associated with dorsal striatal–posterior cingulate connectivity (Wang et al., 2018). Similarly, another recent study by Rössler and colleagues reported ventral striatal dysconnectivity in a schizotypy sample and provided preliminary evidence that this might indeed result from dopaminergic alterations, supporting the dysconnection hypothesis (Rössler et al., 2018). In particular, the authors found lower ventral striatal connectivity in participants who scored higher on a schizotypy scale regardless of whether they had received an L-Dopa or placebo challenge, whereas participants with lower schizotypy scores showed striatal dysconnectivity following L-Dopa administration (Rössler et al., 2018). However, the samples used in both studies above were largely composed of individuals with scores in the low to moderate range. It thus remains unclear whether corticostriatal dysconnectivity extends to individuals with high positive schizotypy scores. This is an important question as previous studies in schizophrenia and CHR subjects indicate that the greater the rs-fMRI dysconnectivity, the higher the severity of positive symptoms (Dandash et al., 2014; Fornito et al., 2013), and that high scores in positive schizotypy scales are associated with higher severity of positive symptoms in patients with schizophrenia (Barrantes-Vidal et al., 2015; Cochrane et al., 2010).

The high schizotypy paradigm is a widely used strategy to examine neurobiological factors related to the expression of psychotic symptoms in the absence of possibly confounding disease-related effects such as antipsychotic medication exposure and illness chronicity which can affect rs-fMRI data (Sarpal et al., 2017, 2015). However, additional confounders in imaging studies may arise from technical limitations, as for example, rs-fMRI data tends to be noisy and may result in indeterminacy of the sources of blood oxygenation level dependent (BOLD) signals, particularly within subcortical regions (Power et al., 2012; Shmueli et al., 2007). Previous studies in psychosis and schizotypy used standard rs-fMRI, which is based on single-echo echo-planar imaging (EPI) sequences employing echo times (TEs) designed to roughly correspond to the average tissue T2*, in order to optimize contrast (Kundu et al., 2012). However, because T2* varies regionally, so does the signal-to-contrast ratio, resulting in signal loss in parts of the brain where T2* is particularly short or long (e.g. Posse et al., 1999). This compromises the quality of the data, especially in low T2* regions such as the inferior temporal cortices, or indeed the orbitofrontal cortex and ventral striatum (Kundu et al., 2017). Adding to this issue, rs-fMRI connectivity findings are highly vulnerable to spurious effects: because they are often based on correlational analyses, any factor that simultaneously influences signal in more than one region of the brain will increase observed connectivity, while factors that influence signal in a single region will decrease observed connectivity, such as head motion, cardiac and respiratory rates, arterial CO₂ concentration and blood pressure (see e.g. Murphy et al., 2013 for a detailed discussion). Typically, this type of so called physiological noise is dealt with using band-pass-filtering for the BOLD signal frequency band (0.01 Hz–0.08 Hz) and removal of the variance explained by separately acquired physiological nuisance recordings using linear regression (Murphy et al., 2013). However, significant noise remains even after data clean-up (Power et al., 2012), and nuisance variation that has not been modelled will inevitably remain. These limitations can be addressed by using an fMRI sequence that collects multiple echoes after each pulse. Firstly, the collection of multiple echoes allows for the relaxometric estimation of region specific T2* values, and hence for the voxel-wise computation of a contrast optimized signal from appropriately weighted echoes, which drastically improves overall contrast-to-noise ratio (Kundu et al., 2017; Posse et al., 1999). Secondly, the collection of multiple echoes allows for blind separation of BOLD-like from non-BOLD-like signal components: while the observed percent signal change Δ*S* always depends on both changes in the initial signal intensity (S_0_) and changes in T2*, BOLD effects modulate T2* much more than S_0_, and non-BOLD effects (e.g. head motion, cerebrovascular pulsatility, hardware related fluctuation etc.) modulate S_0_ much more than T2*. Since T2* scales linearly with TE but S_0_ does not, regression of signal components identified using independent component analysis against TE and S_0_ can be used to differentiate between BOLD- and non-BOLD-like components (Kundu et al., 2017, Kundu et al., 2013, Kundu et al., 2012). Thus, nuisance contributions can be reliably removed even if their source is unknown.

To date, these technical limitations have not been addressed in investigations of rs-fMRI connectivity in the psychosis spectrum. Hence, this study sought to investigate rs-fMRI corticostriatal connectivity in a sample of healthy adults with high scores on a psychometric measure of positive schizotypy using contrast-optimized, and independent components analysis (ICA)-denoised, multi-echo (ME) EPI data. Based on findings implicating corticostriatal dysconnectivity in the emergence of positive psychotic symptoms (Dandash et al., 2014; Fornito et al., 2013; Rössler et al., 2018; Wang et al., 2018), we hypothesized that individuals with high positive schizotypy (HS) would show altered corticostriatal functional connectivity compared to a group of similar individuals with low positive schizotypy (LS) scores as control group.

## 2 Methods and materials

### 2.1 Participants

Two hundred and fifty potential participants who had responded to online advertising via the Research Volunteer Recruitment Webpage of King's College London were pre-screened using the short version of the Oxford-Liverpool Inventory of Feelings and Experiences (O-LIFE) (Mason and Claridge, 2006). Subjects were invited to participate in the study if they scored <2 (low positive schizotypy, LS) or >7 (high positive schizotypy, HS) on the Unusual Experiences subscale of the O-LIFE, as in a previous imaging study in our center (Premkumar et al., 2012). The UE subscale of the O-LIFE questionnaire reflects positive schizotypy and is associated with positive symptoms in schizophrenia patients (Cochrane et al., 2010).

Participants were excluded if they had a history of neurologic/psychiatric disorders as assessed using the Mini International Neuropsychiatric Inventory (Sheehan et al., 1998) and the Psychosis Screening Questionnaire (Bebbington and Nayani, 1995) (administered by a trained interviewer and reviewed by an experienced neuropsychologist, GM). Other exclusion criteria included contraindications to MRI scanning, having a first-degree relative with present/past history of psychotic disorder, present/past history of use of psychotropic medications, and use of recreational drugs in the two weeks prior to scanning or meeting criteria for substance abuse/dependence by self-report. The final sample included 20 participants in both the HS (10 males; age range 18–39 years, M = 26.35, SD = 5.47) and LS groups (11 males; age range 18–44 years, M = 26.70, SD = 7.06). Three studies have reported previous findings from overlapping sub-samples of this cohort with other imaging modalities (Modinos et al., 2018, Modinos et al., 2017a, Modinos et al., 2017b).

Ethical approval for the study was obtained from the KCL College Research Ethics Committee (CREC) and all participants provided written informed consent before initiating any study procedures.

### 2.2 Behavioral assessments

On the day of scanning, before scanning commenced, participants completed a semi-structured interview adapted from the Early Psychosis Prevention and Intervention Centre (EPPIC) Drug and Alcohol Assessment Schedule (http://www.eppic.org.au) to assess current/past medication use and current/past use of alcohol, tobacco and cannabis; the Social Function Questionnaire (SFQ) (Tyrer et al., 2005) to measure social functioning; and a validated short version of the Wechsler Adult Intelligence Scale-III (WAIS- III) (Velthorst et al., 2013) to measure intellectual ability. Analysis of demographic and questionnaire data was performed in SPSS 24 (https://www.ibm.com/analytics/us/en/technology/spss/), with the effect of group being tested using independent sample *t*-tests for parametric data and χ^2^-tests for non-parametric data (significance threshold *p* < .05).

### 2.3 Imaging acquisition

Scanning was performed on a General Electric Discovery MR750 3 T system (Milwaukee, WI, USA) at the Institute of Psychiatry, Psychology & Neuroscience, King's College London. For the rs-fMRI, participants were asked to lie still with their eyes open, and to think of nothing in particular while a fixation cross was displayed in the center of a screen which they viewed through a mirror system. Scanning time for the rs-fMRI was 12 min. During this time, ME-EPI images sensitive to BOLD contrast were acquired to measure hemodynamic responses (repetition time: 2500 ms; echo times, 12, 28, 44, 60 ms; flip angle, 80°; 4.0 × 4.0 × 3.0-mm voxels; field of view, 240; 32 axial sections collected with sequential (top down) acquisition and 1-mm interslice gap). A structural scan was acquired for co-registration of the ME-EPI data by means of a three-dimensional T1-weighted inversion recovery-prepared gradient echo sequence (voxel size: 1.05 × 1.05 × 1.2 mm, field of view: 270 mm, 196 slices, repetition time: 7.3 ms, echo time: 3.0 ms, inversion time: 400 ms).

### 2.4 Imaging preprocessing

After resetting of the origins for both T1-weighted and ME-EP images, a study specific template was created using Advanced Normalization Tools (ANTs; http://stnava.github.io/ANTs/) for later normalization, in order to reduce localization error and improve sensitivity (Huang et al., 2010). One subject from the HS group had to be excluded because of atypical anatomy which undermined the template quality, resulting in a final sample of 20 LS and 19 HS subjects. The ME-EPI echoes were separated into four distinct time series (corresponding to the four individual echoes), which were then de-spiked using 3dDespike in the Analysis of Functional NeuroImages (AFNI) framework (https://afni.nimh.nih.gov), and slice time corrected using SPM12 (http://www.fil.ion.ucl.ac.uk/spm/software/spm12/). Parameters for motion correction were estimated from the first echo, and applied to all four echoes using FSL's mcFLIRT (Greve and Fischl, 2009; Jenkinson et al., 2002; Jenkinson and Smith, 2001). Subjects' ME-EP images were then co-registered to the T1 scan using boundary-based registration as implemented in FLIRT. Again, parameters were estimated for the first echo, and subsequently applied to all four echoes. All echoes were spatially normalized to the study-specific template, and from there to Montreal Neurological Institute (MNI) space. Finally, the images from all echoes were z-concatenated for further processing, i.e. the space-by-time matrices from each echo were appended to one another in the z-direction to form a single matrix using the *3dZcat* function in AFNI. TEDANA, a python script that forms part of the Multi Echo Independent Component Analysis (MEICA) package (https://afni.nimh.nih.gov/pub/dist/src/pkundu/meica.py) (Kundu et al., 2017, Kundu et al., 2013, Kundu et al., 2012) was called to perform TE dependent ICA-based denoising and T2* weighted averaging (optimal combination) of echoes as described above. The denoised, optimally combined images were subsequently taken forward for motion correction, removal of white matter (WM) and cerebrospinal fluid (CSF) signal via regression, and band-pass-filtering (frequency range 0.08–0.01 Hz). A comparison of the mean framewise displacement (FD) (Power et al., 2012) between HS and LS subjects revealed no significant difference in head motion between groups (*t* = 0.358, *p* = .72). No individual subject showed mean FD in excess of 0.12 mm.

### 2.5 Imaging analysis

We defined six bilateral striatal seeds based on previous validated work on striatal connectivity (Dandash et al., 2015, Dandash et al., 2014; Di Martino et al., 2008; Fornito et al., 2013; Rössler et al., 2018; Wang et al., 2018, Wang et al., 2016): ventral striatum inferior/nucleus accumbens (VSi; ±9, 9, −8); ventral striatum superior (VSs; ±10, 15, 0); dorsal caudate (DC; ±13, 15, 9); dorsal caudal putamen (DCP; ±28, 1, 3); dorsal rostral putamen (DRP; ±25, 8, 6); and ventral rostral putamen (VRP; ±20, 12,–3) (Fig. 2A), with the radius set at 3.5 mm (Di Martino et al., 2008). The mean signal was extracted from the seed regions (voxel-wise) using the REST toolbox (Song et al., 2011) to perform Pearson's correlation coefficients between these regressors and the rest of the brain (voxel-wise), which were subsequently Fisher's Z transformed.

The resulting *Z*-maps were then taken to group-level whole-brain analysis using the General Linear Model as implemented in SPM12. Connectivity differences between groups were examined using *t*-contrasts. We used a cluster forming threshold of *p* < .001 uncorrected, to then enforce cluster-wise correction for multiple testing at *p* < .05 family-wise error (FWE) rate, based on previous studies (Dandash et al., 2014; Di Martino et al., 2008; Fornito et al., 2013; Wang et al., 2018). Potential effects of age or substance use (alcohol, cigarettes and cannabis) on areas showing significant group differences in connectivity were examined with an additional ANCOVA in SPM.

Finally, associations between symptom scores (O-LIFE UE) in HS subjects and *Z*-scores averaged across clusters showing group differences were analyzed using linear regression in SPSS.

## 3 Results

### 3.1 Demographic and questionnaire results

Table 1 summarizes the sociodemographic characteristics of each group. HS and LS differed, by design, only on the schizotypy measures. Specifically, HS had higher scores on the O-LIFE subscales measuring unusual experiences, cognitive disorganization, and impulsive non-conformity (all *p* < .001).

**Table 1:** Demographic and questionnaire data.

Characteristic	Low Schizotypy (n = 20)	High Schizotypy (n = 19)	*t*/χ^2^	P
Gender (male/female)	10/10	10/09	0.027	0.869
Age (years)	26.35 ± 5.47	26.37 ± 7.09	−0.009	0.993
Education (years)	17.41 ± 3.75	18.25 ± 5.12	−0.532	0.599
Urbanicity (% urban)	94.1	64.3	5.723	0.057
ESeC social class (% salariat)	63.2	82.4	3.195	0.202
SFQ	4.20 ± 3.22	5.84 ± 2.93	−1.662	0.105
WAIS III	121.85 ± 12.42	119.79 ± 17.49	0.422	0.676
O-LIFE Total	16.16 ± 9.34	39.0 ± 11.82	−6.388	< 0.001
O-LIFE Unusual Experiences	0.75 ± 0.97	11.42 ± 4.31	−9.838	< 0.001
O-LIFE Cognitive Disorganization	5.45 ± 4.56	11.72 ± 6.33	−3.530	0.001
O-LIFE Impulsive Nonconformity	4.05 ± 2.76	8.71 ± 1.99	−5.783	< 0.001
O-LIFE Introvertive Anhedonia	6.11 ± 4.70	6.44 ± 4.03	−0.235	0.816
Daily tobacco use	0.82 ± 3.44	0.35 ± 0.81	0.581	0.565
Daily caffeine use	1.67 ± 1.48	2.82 ± 2.48	−1.777	0.084
Alcohol use (median(range))	2 (0–5)	1 (0–4)	0.648	0.421
Marijuana use (median(range))	1 (0–3) (n = 19)	0 (0–3)	1.727	0.189

Abbreviations: ESeC, European Socio-economic Classification; CTQ, Childhood Trauma Questionnaire; SFQ, Social Function Questionnaire; WAIS III, Wechsler Adult Intelligence Scale III; O-LIFE, Oxford–Liverpool Inventory of Feelings and Experiences; SPQ, Schizotypal Personality Questionnaire. For the Social Functioning Questionnaire, higher scores indicate greater social impairment.

### 3.2 Imaging results

#### 3.2.1 Resting-state fMRI connectivity

**Fig. 1:**
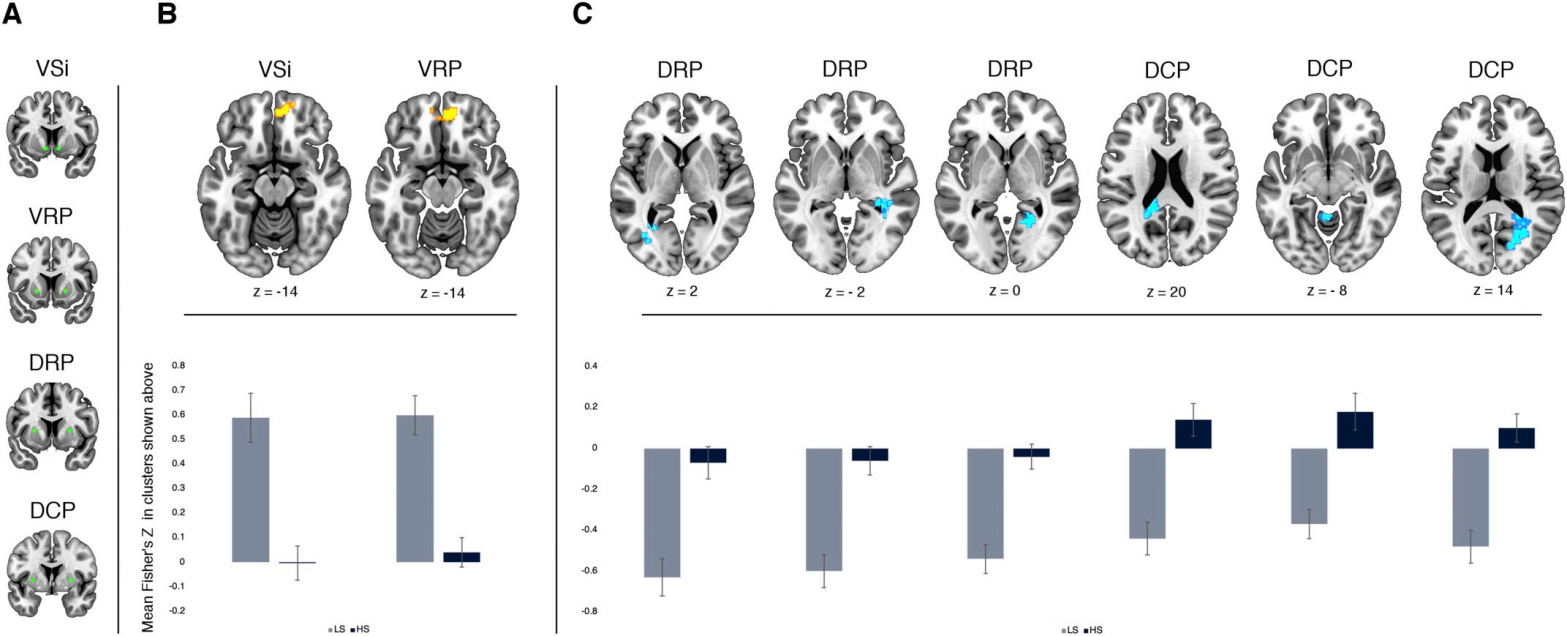
Statistical parametric maps showing (A) relevant seed regions, as well as (B) decreased positive and (C) decreased negative functional connectivity in high (HS) vs low (LS) schizotypy subjects, by seed, and corresponding mean Fisher's *Z*-values by group. Error bars in graphs represent standard error of the mean.

To test our hypothesis that individuals with HS would show altered functional connectivity of the striatum relative to subjects with LS, we compared Fisher's *Z*-values of whole-brain connectivity for each striatal seed between groups. This analysis revealed hypoconnectivity in HS compared to LS individuals between ventral striatal regions and the ventromedial PFC. Specifically, hypoconnectivity was observed between the VSi and a cluster including the bilateral gyrus rectus and right medial orbital gyrus (cluster-wise p_FWE_ = 0.037), and between the VRP a cluster including the right medial orbital gyrus, left gyrus rectus and right anterior cingulate cortex (cluster-wise p_FWE_ < 0.001) (Table 2, Fig. 1B).

**Table 2:** Differences in Fisher's Z-values for resting-state fMRI striatal connectivity between high and low schizotypy.

Seed	Area	Side	MNI coordinates			Z	K_E_	*p*_FWE_
			x	y	z			Cluster
*LS* > *HS*								
VSi	Gyrus rectus	R	10	50	−18	4.01	64	0.037
	Medial Orbital Gyrus	R	2	52	−14	3.54		
	Frontopolar cortex	R	18	58	−14	3.27		
VRP	Medial Orbital Gyrus	R	8	48	−14	4.85	172	0.000
	Gyrus rectus	L	−10	54	−18	4.23		
	Anterior Cingulate Cortex	R	6	42	−2	3.89		
*LS* < *HS*								
DRP	Hippocampus	R	32	−38	−2	3.98	98	0.003
	Middle Temporal Gyrus	R	40	−48	4	3.79		
	Hippocampus	R	40	−34	−6	3.70		
	Calcarine Sulcus	R	34	−56	8	3.97	151	0.000
	Middle Occipital Gyrus	L	−28	−60	2	3.91	90	0.005
	Middle Occipital Gyrus	L	−32	−70	4	3.71		
	Calcarine Sulcus	L	−32	−58	10	3.56		
DCP	Middle Occipital Gyrus	R	30	−60	20	4.35	488	0.000
	Calcarine Sulcus	R	28	−60	4	4.38		
	Calcarine Sulcus	R	28	−50	8	4.26		
	Posterior Cingulate	L	−18	−46	20	4.25	293	0.000
	Hippocampus	L	−30	−40	2	4.16		
	Calcarine Sulcus	L	−28	−56	6	3.93		
	Cerebellum	L	−2	−50	−8	4.01	64	0.038
	Culmen	L	−6	−60	−2	3.72		

Results are considered significant at cluster-wise *p*_FWE_ < 0.05.

DC, dorsal caudate; DCP, dorsocaudal putamen; DRP, dorsorostral putamen; FWE, family-wise error; HS, high schizotypy; *k*_E_, cluster extent. L, left; LS, low schizotypy; R, right; VRP, ventrorostral putamen; VSi, ventral caudate inferior; VSs, ventral caudate superior.

Furthermore, we found hypoconnectivity between dorsal striatal regions and temporo-occipital areas in HS compared to LS subjects. More specifically, HS subjects showed hypoconnectivity between the DRP and a cluster centered on the right hippocampus (cluster-wise p_FWE_ < 0.001) extending into occipital regions, left middle occipital gyrus (cluster-wise p_FWE_ = 0.005), and calcarine sulcus (cluster-wise p_FWE_ = 0.001); and between the DCP and the right middle occipital gyrus/calcarine sulcus (cluster-wise p_FWE_ < 0.001), the left hippocampus (cluster-wise p_FWE_ < 0.001), and cerebellar areas (cluster-wise p_FWE_ = 0.038) (Table 2, Fig. 1C).

**Fig. 2:**
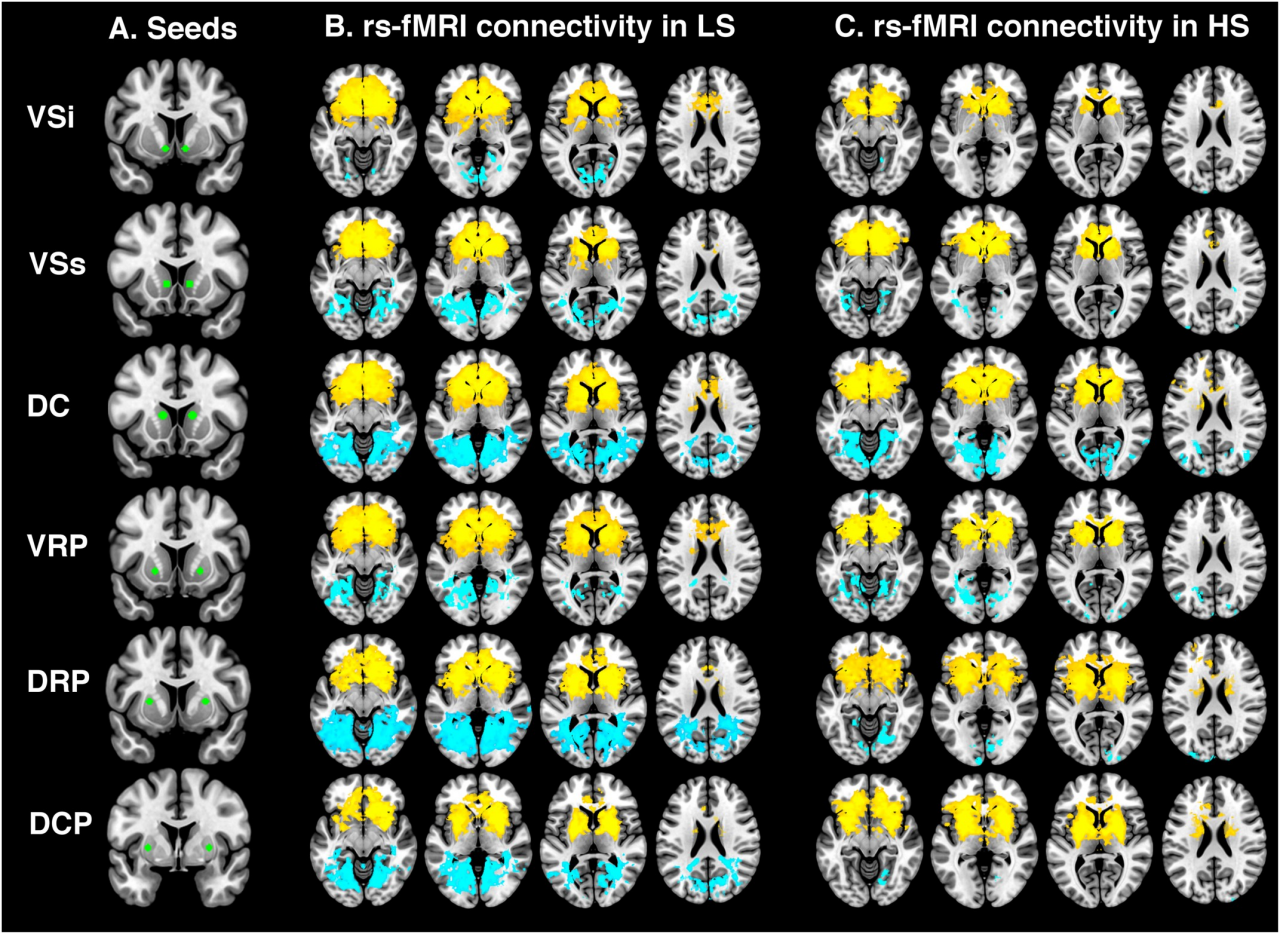
(A) Location of striatal seed regions (green). Within-group functional connectivity maps between each striatal seed region and the whole brain in (B) LS subjects and (C) HS subjects. Blue refers to negative coupling, yellow refers to positive coupling. Maps thresholded at *p* < .001 uncorrected for display purposes. DC, dorsal caudate; DCP, dorsocaudal putamen; DRP, dorsorostral putamen; VRP, ventrorostral putamen; VSi, ventral caudate inferior; VSs, ventral caudate superior.

There were no other regions showing significant differences in rs-fMRI. For completeness, within-group rs-fMRI connectivity results for each striatal seed and the rest of the brain are shown in Fig. 2B and C. For a full list of significant clusters within each group, see Supplementary Table 1.

#### 3.2.2 Effect of potential confounders

Groups were matched in demographic variables and including age as a covariate of no interest in the imaging analysis did not change the results. The hypoconnectivity between between DRP – calcarine sulcus, DCP – hippocampus, and DCP – middle occipital gyrus remained apparent at cluster-wise p_FWE_ < 0.05 when alcohol, cigarette and cannabis use were added to the statistical model as covariates of no interest. However, adding these variables as covariates of no interest rendered the reductions in VSi – vmPFC, VRP – vmPFC, DCP-cerebellum and DRP-middle occipital gyrus connectivity no longer significant at cluster-wise p_FWE_ < 0.05.

#### 3.2.3 Associations with schizotypy scores

Within HS subjects, linear regression of *Z*-scores averaged across significant clusters against O-LIFE UE scores revealed no significant associations with either ventral or dorsal striatal connectivity (all *p* > .05). However, there were trend-level positive associations between positive schizotypy scores as assessed by O-LIFE UE and VSi-vmPFC and VRP-vmPFC connectivity, respectively (F(17,1) = 4.046, *p* = .061, R^2^ = 0.152; and F(17,1) = 4.403, *p* = .052, R^2^ = 0.167).

In an exploratory analysis, we further assessed the association between significant clusters and the O-LIFE subscores Impulsive Non-conformity and Cognitive Disorganization in HS subjects. Linear regression revealed no significant associations with either ventral or dorsal striatal connectivity (all *p* > .05). Because the O-LIFE subscore Introvertive Anhedonia did not differ between groups, we tested the association between this subscore and connectivity indices across both HS and LS. Again, linear regression revealed no significant associations with either ventral or dorsal striatal connectivity (all *p* > .05).

## 4 Discussion

Using novel multi-echo rs-fMRI methodology, the present study found lower corticostriatal resting-state functional connectivity in healthy individuals with high levels of psychotic-like experiences compared to those without such experiences (LS). These findings support the notion that corticostriatal dysconnectivity is involved in the expression of psychotic-like experiences across the extended psychosis phenotype, including healthy individuals, people at CHR of psychosis, and patients with an established psychotic disorder (Dandash et al., 2014; Fornito et al., 2013; Rössler et al., 2018; Wang et al., 2018). Interestingly, rs-fMRI striatal connectivity in HS subjects was characterized by both a lower level of positive coupling and a lower level of negative coupling compared to LS subjects. Specifically, distinct functional connectivity patterns were detected along the ventral-dorsal striatal axis; HS subjects showed lower positive coupling between the ventral striatum and vmPFC, and lower negative coupling between the dorsal striatum and temporo-occipital regions including the hippocampus, middle occipital gyrus and calcarine sulcus.

Previous studies have shown that striatal dysconnectivity in patients with schizophrenia (Fornito et al., 2013), CHR subjects (Dandash et al., 2014) and individuals with low-to-moderate levels of positive schizotypy (Rössler et al., 2018; Wang et al., 2018) may be characterized by both higher and lower connectivity of different striatal regions. This pattern has been proposed to indicate a potential risk biomarker for psychosis onset in vulnerable individuals (Dandash et al., 2014). However, the directionality of the changes in previous studies has shown some inconsistency; the above studies reported a dorsal-to-ventral gradient of hypoconnectivity to hyperconnectivity with frontal regions (Dandash et al., 2014; Fornito et al., 2013; Wang et al., 2018). In contrast, reduced connectivity between ventral striatal and ventral prefrontal areas has been reported in unmedicated patients with schizophrenia (Lin et al., 2018), in line with the results of the present study in high positive schizotypy. To our knowledge, there are no previous reports of hypoconnectivity between dorsal striatal areas and temporo-parietal regions in patients that would mirror our findings in high schizotypes, suggesting a lack of continuity of this phenotype across the psychosis spectrum (e.g., Fornito et al., 2013; Lin et al., 2018). Further, we found no evidence of an association between dorsal system alterations and symptom scores in our data. We are therefore cautious to interpret our finding as a reflection of psychotic symptoms. A possible explanation is that, rather than a pathological mechanism, the group difference might reflect a resilience mechanism that protects healthy individuals with high psychometric schizotypy from their psychotic experiences to become clinically relevant.

Previous work on schizotypy has reported striatal hypoconnectivity with posterior regions (albeit with ventral striatal regions) (Rössler et al., 2018), but lower connectivity with the vmPFC has not been reported (Rössler et al., 2018; Wang et al., 2018). These discrepancies may relate to the nature of the sample, as we used a group of subjects comprising high scorers in positive schizotypy as identified using a measure of subclinical psychotic experiences such as the O-LIFE, while Rössler et al. (2018) and Wang et al. (2018) studied low-to-moderate scorers as identified using the Schizotypal Personality Disorder Questionnaire (SPQ). The differences may also relate to the application of a multi-echo rs-fMRI sequence in our study but not in Rössler et al.'s (2018) and Wang et al.'s (2018) studies, such that our finding of lower vmPFC-ventral striatal connectivity in the HS group may have been masked in those studies since these regions have low CNR in standard EPI-sequences (Kundu et al., 2017).

While we did not detect significant associations between connectivity differences and positive schizotypy scores in the HS group, there was trend level evidence for a positive relationship between ventral striatum – vmPFC connectivity and the Unusual Experiences scale of the O-LIFE. Due to the limited size (*N* = 19) and low variability along the schizotypy dimension within the HS group, the possibility that there was insufficient power to detect significant associations with UE scores cannot be ruled out. Nevertheless, our findings highlight the promising applicability of multi-echo rs-fMRI methods for the detection of dysconnectivity patterns in a cross-sectional study design. Further research assessing functional connectivity across different groups across the psychosis spectrum (healthy individuals with low and high schizotypy, CHR subjects and unmedicated first-episode psychosis patients) with larger sample sizes will help clarify whether rs-fMRI connectivity changes vary according to the degree of vulnerability and of severity of psychotic experiences.

Mechanistically, the dysconnection hypothesis of psychosis proposes that the likely neurobiological basis for dysconnectivity would be aberrant neuromodulation (Friston et al., 2016). Animal work suggests that altered striatal dopaminergic signaling may disrupt the ventral aspects of frontostriatal connectivity in relation to psychosis phenotypes as, for example, in the rodent nucleus accumbens, mPFC afferents are modulated by dopamine via D2 receptors, such that increases in tonic dopamine levels attenuate mPFC inputs (Grace et al., 2007). In this context, ventral striatal-vmPFC hypoconnectivity in positive schizotypy could be driven by increased tonic striatal dopamine (Howes et al., 2012). Indeed, there is some evidence of an association between schizotypy scores, disrupted dopaminergic neurotransmission, and striatal dysconnectivity (Rössler et al., 2018; Woodward et al., 2011), although findings have been less consistent than in frank psychosis, possibly due to high heterogeneity in the experimental designs and methods used (Mohr and Ettinger, 2014).

An interesting corollary of the hypothesis that a dopaminergic dysfunction of the striatum in the psychosis spectrum compromises the functional integrity of the limbic cortico-striatal loop is that it might also account for reduced vmPFC connectivity with the default mode network (DMN) in psychosis patients (Camchong et al., 2011; Hu et al., 2017). Striatal dopamine function has been directly associated with vmPFC-DMN connectivity in a study of the effects of antipsychotics on the DMN (Sambataro et al., 2010), as well as in an investigation of a single nucleotide polymorphism of the D2 receptor gene (Sambataro et al., 2013). Consistent with a disruption of coordinated activity of the vmPFC driven by putatively dopaminergic striatal abnormalities, Wang et al. report a breakdown of the reciprocal interaction between the striatum and DMN nodes, including the vmPFC, in schizophrenia (Wang et al., 2015a, Wang et al., 2015b).

An alternative mechanistic explanation could be that connectivity alterations relate to changes in GABA- or glutamatergic alterations in the striatum, or indeed that primary vmPFC dysfunction may lead to dysconnectivity, as preclinical evidence suggests that striatal hyperdopaminergia may be a downstream effect of a failure of the mPFC to regulate hippocampal hyperresponsivity (Gomes and Grace, 2017; Grace, 2016; Patton et al., 2013; Zimmerman and Grace, 2016). Consistent with this notion, our recent positive schizotypy work found increased resting-state perfusion of the hippocampus in a largely overlapping sample (Modinos et al., 2018), in line with previous CHR studies (Allen et al., 2017, Allen et al., 2016; Schobel et al., 2013).

In this study, we extend the existing knowledge by investigating resting-state connectivity in otherwise healthy individuals on the high end of the schizotypy spectrum using multi-echo fMRI data. Our approach is advantageous for two reasons: First, the acquisition of multiple echoes is thought to afford superior noise removal and contrast optimization compared to traditional techniques, yielding better quality data. Second, variation due to illness chronicity, medication status, impaired global function and other illness associated factors are curtailed as in other studies on schizotypy, but high schizotypes are arguably phenotypically closer to psychosis patients that those in the low to moderate range that were previously examined (Rössler et al., 2018; Wang et al., 2018). Thus, our results are an important addition to the literature on the role of striatal dysconnectivity in the development and maintenance of psychotic traits.

One limitation of the present study is that the cross-sectional nature of the study prevents elucidating whether the observed findings reflect a risk or a resilience phenotype, in particular given reports that high schizotypes have a lower likelihood of developing psychosis than a CHR group (Kwapil et al., 2013). Further, while participants were asked to refrain from taking recreational drugs for two weeks prior to scanning, obtaining a biological measure of drug use on the day of scanning would have helped further confirm this issue (e.g., urine sample). The ventral striatal – vmPFC and dorsal striatal-occipital hypoconnectivity were no longer significant after including substance use (alcohol, tobacco and cannabis) in the statistical analysis. Finally, our study aimed at elucidating the role of striatal connectivity in the expression of psychotic-like experiences of the positive dimension based on previous literature in patients with psychosis and animal models, but further studies including schizotypal individuals based on negative and disorganized dimensions would clarify whether differences in functional connectivity are also related to negative and disorganized traits.

Future work with multi-echo rs-fMRI should directly investigate how striatal connectivity differences relate to markers of striatal dopaminergic function across the extended psychosis phenotype in patients, high-risk individuals, and high schizotypes. This would provide crucial evidence regarding the similarities and differences in striatal connectivity between individuals across the spectrum, yielding clues as to the potential determinants of psychosis risk, the occurrence of symptoms, illness-status and severity. Additionally, longitudinal studies should investigate which connectivity changes accompany transition to frank psychosis, as well as potential protective factors. Further, the functional and behavioral consequences of aberrant connectivity in psychosis spectrum disorders should be ascertained in studies combining resting state and functional measures of e.g. salience attribution.

### 4.1 Conclusion

Using a multi echo rs-fMRI sequence and independent component analysis, we found that high compared to low positive schizotypy was associated with lower functional connectivity between ventral aspects of the striatum and the vmPFC and between dorsal striatal regions and temporo-occipital areas. Given that aberrant functional integration has been implicated in the pathophysiology of psychosis, the present results offer some support to the notion of a central role of striatal dysconnectivity in the extended psychosis spectrum.

The following is the supplementary data related to this article.

Supplementary Table 1 Resting-state fMRI striatal connectivity in high and low schizotypy. Supplementary Table 1

## Declaration of interest

GJB received honoraria for teaching from General Electric Healthcare, and acted as a consultant for IXICO, at the time of this study. The other authors declare no competing financial interests.
