# Supplementary material for: Multi-echo fMRI, resting-state connectivity, and high psychometric schizotypy
Source: Neuroimage Clin. 2018 Nov 20;21:101603. doi: 10.1016/j.nicl.2018.11.013 (PMC6413302; doi:10.1016/j.nicl.2018.11.013)
Supplement: Supplementary Table 1 — Resting-state fMRI striatal connectivity in high and low schizotypy. [file mmc1.docx]

| **Supplementary Table 1.** Resting-state fMRI striatal connectivity in high and low schizotypy. | | | | | | |
| --- | --- | --- | --- | --- | --- | --- |
| **Group** | **Seed** | **Positive/negative** | **MNI coordinates** | ***k*** | **Z** | ***p*_FWE (cluster)_** |
| LS | VSi | positive | -10 10 -8 | 17307 | 7.80 | 0.000 |
|  |  |  | 12 10 -6 |  | 7.38 |  |
|  |  |  | 10 18 -4 |  | 7.08 |  |
|  |  |  | -4 -6 30 | 101 | 4.55 | 0.003 |
|  |  |  | 2 -12 30 |  | 3.98 |  |
|  |  |  | -8 -6 22 |  | 3.48 |  |
|  |  | negative | 12 -74 16 | 1422 | 4.94 | 0.000 |
|  |  |  | 12 -66 10 |  | 4.76 |  |
|  |  |  | 10 -74 8 |  | 4.61 |  |
|  |  |  | 2 -64 -28 | 125 | 4.00 | 0.001 |
|  |  |  | 0 -54 -36 |  | 3.77 |  |
|  |  |  | -4 -66 -36 |  | 3.55 |  |
|  | VSs | positive | -6 14 -2 | 11570 | 7.05 | 0.000 |
|  |  |  | 12 16 -12 |  | 7.04 |  |
|  |  |  | 12 16 -2 |  | 6.89 |  |
|  |  | negative | 20 -52 0 | 5687 | 5.22 | 0.000 |
|  |  |  | -28 -58 -2 |  | 5.16 |  |
|  |  |  | -30 -50 -8 |  | 4.74 |  |
|  |  |  | -8 -46 -20 | 334 | 4.23 | 0.000 |
|  |  |  | 20 -36 -22 |  | 4.19 |  |
|  |  |  | -6 -52 -26 |  | 4.19 |  |
|  |  |  | 8 -76 -32 | 176 | 4.20 | 0.000 |
|  |  |  | 2 -70 -28 |  | 4.13 |  |
|  |  |  | -10 -64 -32 |  | 3.58 |  |
|  |  |  | 38 -18 42 | 65 | 4.09 | 0.031 |
|  |  |  | 50 -16 42 |  | 3.67 |  |
|  | DC | positive | -20 18 4 | 17134 | 6.76 | 0.000 |
|  |  |  | -14 14 -16 |  | 6.75 |  |
|  |  |  | -20 26 0 |  | 6.75 |  |
|  |  | negative | 22 -54 -2 | 13037 | 6.05 | 0.000 |
|  |  |  | 10 -58 0 |  | 5.56 |  |
|  |  |  | 14 -46 0 |  | 5.53 |  |
|  |  |  | 0 -42 58 | 159 | 4.29 | 0.000 |
|  |  |  | 12 -46 56 |  | 4.06 |  |
|  |  |  | 2 -34 60 |  | 3.74 |  |
|  |  |  | 56 -32 26 | 139 | 4.24 | 0.000 |
|  |  |  | 52 -30 14 |  | 4.05 |  |
|  |  |  | 60 -36 34 |  | 3.53 |  |
|  |  |  | 44 -18 44 | 68 | 3.80 | 0.021 |
|  |  |  | 52 -16 42 |  | 3.48 |  |
|  | VRP | positive | -12 16 -8 | 17805 | 7.69 | 0.000 |
|  |  |  | 22 12 2 |  | 7.62 |  |
|  |  |  | -22 12 4 |  | 7.49 |  |
|  |  | negative | -38 -48 -10 | 2727 | 5.03 | 0.000 |
|  |  |  | -14 -44 -2 |  | 4.88 |  |
|  |  |  | -26 -64 -2 |  | 4.80 |  |
|  |  |  | 34 -52 -14 | 1336 | 4.75 | 0.000 |
|  |  |  | 20 -50 0 |  | 4.75 |  |
|  |  |  | 22 -48 18 |  | 4.73 |  |
|  |  |  | -10 -52 -34 | 66 | 4.17 | 0.033 |
|  |  |  | 30 -56 -36 | 72 | 3.96 | 0.021 |
|  |  |  | 24 -58 -42 |  | 3.91 |  |
|  |  |  | 30 -60 -28 |  | 3.60 |  |
|  | DRP | positive | 24 8 6 | 13603 | 6.98 | 0.000 |
|  |  |  | -26 8 6 |  | 6.41 |  |
|  |  |  | 26 2 -8 |  | 6.34 |  |
|  |  | negative | 28 -68 -6 | 19911 | 6.01 | 0.000 |
|  |  |  | 14 -64 4 |  | 5.91 |  |
|  |  |  | 12 -44 -24 |  | 5.86 |  |
|  |  |  | 0 -42 60 | 82 | 4.45 | 0.009 |
|  |  |  | 2 -34 62 |  | 3.68 |  |
|  |  |  | -10 -34 66 |  | 3.51 |  |
|  | DCP | positive | 30 0 2 | 9676 | 7.16 | 0.000 |
|  |  |  | 30 8 4 |  | 6.72 |  |
|  |  |  | -26 0 4 |  | 6.68 |  |
|  |  | negative | 10 -44 2 | 9713 | 5.63 | 0.000 |
|  |  |  | 14 -62 -2 |  | 5.49 |  |
|  |  |  | -18 -46 20 |  | 5.35 |  |
|  |  |  | 2 -42 -44 | 430 | 4.63 | 0.000 |
|  |  |  | 12 -46 -42 |  | 4.43 |  |
|  |  |  | 28 -48 -42 |  | 4.22 |  |
|  |  |  | -12 -42 -26 | 132 | 4.31 | 0.000 |
|  |  |  | -6 -50 -26 |  | 3.58 |  |
|  |  |  | -4 -34 -22 |  | 3.48 |  |
|  |  |  | -20 -62 -38 | 85 | 4.03 | 0.008 |
|  |  |  | -10 -70 -34 |  | 3.88 |  |
|  |  |  | -14 -56 -44 |  | 3.62 |  |
|  |  |  | -16 -88 42 | 65 | 4.00 | 0.035 |
|  |  |  | -8 -80 38 |  | 3.61 |  |
|  |  |  | 2 -82 38 |  | 3.45 |  |
| HS | VSi | positive | -10 10 -8 | 7150 | 7.67 | 0.000 |
|  |  |  | 12 10 -6 |  | 7.35 |  |
|  |  |  | 10 18 -12 |  | 6.57 |  |
|  |  | negative | -10 -102 16 | 74 | 5.01 | 0.017 |
|  |  |  | -8 -94 28 |  | 3.71 |  |
|  |  |  | 12 -102 14 | 80 | 4.73 | 0.011 |
|  |  |  | 24 -94 16 |  | 3.89 |  |
|  | VSs | positive | -6 14 -2 | 10257 | 7.19 | 0.000 |
|  |  |  | 10 18 -12 |  | 7.06 |  |
|  |  |  | 12 16 0 |  | 7.04 |  |
|  |  | negative | 28 -42 -34 | 976 | 5.10 | 0.000 |
|  |  |  | 26 -54 -42 |  | 4.89 |  |
|  |  |  | 32 -62 -40 |  | 4.55 |  |
|  |  |  | -32 -62 -6 | 597 | 4.47 | 0.000 |
|  |  |  | -38 -58 0 |  | 4.47 |  |
|  |  |  | -30 -50 -18 |  | 4.30 |  |
|  |  |  | 32 -88 26 | 82 | 4.39 | 0.009 |
|  |  |  | 26 -94 28 |  | 4.13 |  |
|  |  |  | 26 -82 30 |  | 3.98 |  |
|  |  |  | -22 -44 -40 | 218 | 4.37 | 0.000 |
|  |  |  | -30 -54 -36 |  | 4.31 |  |
|  |  |  | -20 -64 -36 |  | 4.25 |  |
|  |  |  | 30 -80 -34 | 68 | 4.02 | 0.025 |
|  |  |  | 16 -72 -42 |  | 3.80 |  |
|  |  |  | 26 -72 -42 |  | 3.71 |  |
|  |  |  | 16 -72 42 | 82 | 3.93 | 0.009 |
|  |  |  | 22 -84 48 |  | 3.91 |  |
|  | DC | positive | -14 16 10 | 13290 | 7.30 | 0.000 |
|  |  |  | 18 16 12 |  | 6.76 |  |
|  |  |  | 12 20 -4 |  | 6.69 |  |
|  |  |  | -6 38 44 | 158 | 4.55 | 0.000 |
|  |  |  | 2 40 40 |  | 3.96 |  |
|  |  |  | -8 30 40 |  | 3.70 |  |
|  |  |  | -16 58 26 | 62 | 4.49 | 0.034 |
|  |  | negative | -22 -50 -8 | 5990 | 6.12 | 0.000 |
|  |  |  | -32 -52 -18 |  | 5.65 |  |
|  |  |  | -16 -66 -12 |  | 5.58 |  |
|  |  |  | 12 -44 52 | 319 | 5.11 | 0.000 |
|  |  |  | 4 -52 54 |  | 5.09 |  |
|  |  |  | 14 -34 48 |  | 4.10 |  |
|  |  |  | -26 -46 -30 | 139 | 4.71 | 0.000 |
|  |  |  | -14 -58 -42 |  | 4.08 |  |
|  |  |  | -24 -46 -44 |  | 3.88 |  |
|  |  |  | 4 -94 16 | 76 | 4.66 | 0.012 |
|  |  |  | 64 -52 8 | 59 | 4.59 | 0.044 |
|  |  |  | 52 -56 12 |  | 3.70 |  |
|  |  |  | -44 -74 20 | 184 | 4.53 | 0.000 |
|  |  |  | -40 -82 20 |  | 4.31 |  |
|  |  |  | -40 -84 38 |  | 4.17 |  |
|  |  |  | 42 -72 10 | 68 | 4.39 | 0.021 |
|  |  |  | -50 -70 12 | 69 | 4.14 | 0.020 |
|  |  |  | -50 -80 14 |  | 3.95 |  |
|  |  |  | -44 -64 16 |  | 3.40 |  |
|  |  |  | 0 -78 -32 | 94 | 4.06 | 0.003 |
|  |  |  | 12 -74 -36 |  | 3.72 |  |
|  |  |  | 6 -68 -34 |  | 3.65 |  |
|  |  |  | 44 -74 24 | 66 | 4.03 | 0.025 |
|  |  |  | 38 -66 22 |  | 3.40 |  |
|  | VRP | positive | 20 16 -2 | 8700 | 6.84 | 0.000 |
|  |  |  | -20 14 -2 |  | 6.61 |  |
|  |  |  | 16 18 -12 |  | 6.45 |  |
|  |  | negative | 16 -98 14 | 1025 | 5.17 | 0.000 |
|  |  |  | 24 -92 16 |  | 4.62 |  |
|  |  |  | 10 -82 -2 |  | 4.42 |  |
|  |  |  | -24 -40 -16 | 2658 | 4.83 | 0.000 |
|  |  |  | -18 -76 0 |  | 4.82 |  |
|  |  |  | -22 -62 -36 |  | 4.81 |  |
|  |  |  | -4 66 -12 | 120 | 4.22 | 0.001 |
|  |  |  | 2 64 -6 |  | 4.14 |  |
|  |  |  | -10 64 -4 |  | 3.56 |  |
|  |  |  | -14 -98 10 | 69 | 3.99 | 0.027 |
|  |  |  | -20 -94 4 |  | 3.61 |  |
|  |  |  | -16 -76 26 | 118 | 3.91 | 0.001 |
|  |  |  | -22 -56 20 |  | 3.74 |  |
|  |  |  | -28 -62 22 |  | 3.57 |  |
|  | DRP | positive | 24 8 6 | 14224 | 7.79 | 0.000 |
|  |  |  | -26 8 6 |  | 7.62 |  |
|  |  |  | 28 8 -6 |  | 6.61 |  |
|  |  |  | -12 10 42 | 116 | 4.45 | 0.001 |
|  |  |  | -2 12 46 |  | 4.22 |  |
|  |  |  | -6 16 36 |  | 3.41 |  |
|  |  |  | 12 20 32 | 60 | 3.85 | 0.048 |
|  |  |  | 2 26 38 |  | 3.53 |  |
|  |  |  | 14 14 40 |  | 3.22 |  |
|  |  | negative | 16 -98 14 | 829 | 5.38 | 0.000 |
|  |  |  | 28 -96 12 |  | 4.71 |  |
|  |  |  | 6 -84 -6 |  | 4.68 |  |
|  |  |  | -8 -100 -2 | 91 | 4.68 | 0.005 |
|  |  |  | -16 -100 4 |  | 3.45 |  |
|  |  |  | -6 -102 18 | 97 | 4.47 | 0.003 |
|  |  |  | 0 -96 24 |  | 4.44 |  |
|  |  |  | 0 -90 32 |  | 3.73 |  |
|  |  |  | 26 -90 36 | 116 | 4.40 | 0.001 |
|  |  |  | 20 -86 44 |  | 3.93 |  |
|  |  |  | 24 -84 28 |  | 3.56 |  |
|  |  |  | -30 -92 22 | 117 | 4.27 | 0.001 |
|  |  |  | -40 -84 38 |  | 3.98 |  |
|  |  |  | -32 -88 34 |  | 3.89 |  |
|  |  |  | -10 -82 -10 | 95 | 4.16 | 0.004 |
|  |  |  | -18 -80 -2 |  | 3.88 |  |
|  |  |  | -2 -88 -8 |  | 3.74 |  |
|  |  |  | 44 -78 38 | 62 | 4.15 | 0.041 |
|  |  |  | 38 -80 44 |  | 4.11 |  |
|  |  |  | 46 -80 30 |  | 3.71 |  |
|  | DCP | positive | 30 8 -6 | 14263 | 7.51 | 0.000 |
|  |  |  | 26 2 2 |  | 7.36 |  |
|  |  |  | -28 2 2 |  | 7.26 |  |
|  |  | negative | 16 -102 14 | 73 | 4.97 | 0.019 |
|  |  |  | 28 -94 26 |  | 3.90 |  |
|  |  |  | 28 -98 18 |  | 3.64 |  |
|  |  |  | 20 -66 -4 | 191 | 4.44 | 0.000 |
|  |  |  | 22 -58 -12 |  | 4.10 |  |
|  |  |  | 20 -72 -12 |  | 3.89 |  |
